# The Impact of High-Flow Nasal Oxygen Therapy on Swallowing Function and Aspiration in Patients and Healthy Adults: A Scoping Review

**DOI:** 10.7759/cureus.75287

**Published:** 2024-12-07

**Authors:** Kan Sugishima, Hideaki Sakuramoto, Yusuke Oyama, Akira Ouchi, Kentaro Kaneko, Takuto Fukunaga, Michiko Uchi, Gen Aikawa

**Affiliations:** 1 Dapartment of Nursing, Kurume University Hospital, Kurume, JPN; 2 Department of Critical care and Disaster Nursing, Japanese Red Cross Kyushu International College of Nursing, Munakata, JPN; 3 Department of Nursing, Nagasaki University Graduate School of Biomedical Sciences, Nagasaki, JPN; 4 Department of Adult Health Nursing, College of Nursing, Ibaraki Christian University, Hitachi, JPN; 5 Department of Nursing, School of Nursing, Miyagi University, Kurokawa, JPN; 6 Department of Emergency and Critical Care Medicine, Toho University Hospital, Omori, JPN; 7 Department of Nursing, NHO Ibarakihigashi National Hospital, Tokaimura, JPN; 8 College of Nursing, Kanto Gakuin University, Yokohama, JPN

**Keywords:** aspiration, dysphagia, evaluation of swallowing, high-flow nasal oxygen therapy, swallowing function

## Abstract

High-flow nasal oxygen therapy (HFNO) is highly versatile and employed in varied situations, including after extubation, in cases of respiratory failure, and at the end of life. However, its impact on swallowing function is not yet elucidated. Therefore, this scoping review aimed to clarify how HFNO affects swallowing function and whether it poses a risk for aspiration pneumonia. We searched the databases MEDLINE via PubMed, CINAHL, Web of Science, and Cochrane Central Register of Controlled Trials (CENTRAL) from inception till June 5, 2024, to gather relevant studies. No language restrictions were applied. The eligibility criteria were as follows: (1) studies involving adults using HFNO, (2) studies examining swallowing function and the occurrence of pneumonia, and (3) excluding gray literature such as conference proceedings.

A total of 1449 articles were initially identified, of which 12 that met the inclusion criteria were included in the final analysis. Of them, five involved healthy adults, whereas seven involved patients. Six studies investigated the effects of flow rate on the swallowing function, five studies on healthy adults, and one on patients. The review findings indicated that as the flow rate increased, the swallowing function was affected by the shortening of the latency time of the swallowing response and laryngeal vestibular closure time. Additionally, the increase in the flow rate caused modulation of the swallowing-breathing coordination. However, none of the studies reported that HFNO increases the incidence of pneumonia. The increased flow rates of HFNO affect the swallowing function; however, the actual impact on patients is currently unknown. This study involved a small number of healthy adults; therefore, further research based on the patient characteristics is warranted.

## Introduction and background

High-flow nasal oxygen therapy (HFNO) is widely used in patients with acute respiratory failure [1], post-extubation [2], and in palliative care [3]. HFNO is a type of oxygen therapy that delivers heated and humidified oxygen at a high flow rate (maximum rate: 60 L/min) through a nasal cannula [4]. It has several beneficial physiological effects including the accurate delivery of the set fraction of inspired oxygen (FiO_2_), washout of anatomical dead space, reduction in breathing effort, increased airway pressure, and patient comfort [4]. Moreover, HFNO potentially allows oral feeding during treatment. However, the long-term effects of HFNO are currently unknown. The European Society of Intensive Care Medicine (ESICM) guidelines state that cognitive, functional, and quality-of-life issues must be considered to determine the long-term outcomes of HFNO in acute respiratory failure [5].

Several systematic reviews and meta-analyses have investigated the effects of HFNO. A previous study showed no beneficial effect on severe desaturation, serious complications, and overall survival during the peri-intubation period [6]. In contrast, HFNO significantly reduces post-extubation respiratory failure compared with conventional oxygenation therapy [7] and is as effective as non-invasive intermittent ventilation (NIV) in post-extubation respiratory support [8]. However, few studies have explored the relationship between HFNO and the swallowing function.

Dysphagia following extubation in critically ill patients is a serious issue [9] and it is critical to assess the impact of HFNO on swallowing function. Recently, numerous studies have evaluated the impact of HFNO on swallowing function in healthy participants and patients [10-12]. However, the available evidence is limited, and the effects of HFNO on swallowing function are not elucidated. Therefore, we conducted a scoping review aimed at collecting and analyzing evidence on this topic by examining how HFNO affects swallowing and what measures are effective. We believe this will help establish and standardize measures for the safe care of patients with HFNO.

This review aimed to review the relevant literature on the frequency of dysphagia due to HFNO, assessment methods, and pneumonia associated with HFNO in adult patients and healthy adults, and to identify the available evidence.

## Review

Methods

Registration and Search Strategy

The formulated research questions were as follows: “How does HFNO affect the swallowing function?” and “Is HFNO a risk factor for aspiration pneumonia?” This review was conducted per the Preferred Reporting Items for Systematic Reviews and Meta-Analyses Extension for Scoping Reviews (PRISMA-ScR) guidelines (see Appendices) [13]. The review protocol has been registered with the Open Science Framework (https://osf.io/jm4ar/).

A preliminary search was conducted using PubMed alone, through which key terms for the search were identified after screening titles and abstracts. We searched the following databases from inception to June 5, 2024: MEDLINE via PubMed, CINAHL, Web of Science, and Cochrane Central Register of Controlled Trials (CENTRAL). No language restrictions were imposed, and the complete search strings are presented in Table 1.

**Table 1:** Search strategy

Database	Search terms
PubMed	("high flow nasal cannula oxygen therapy” [tiab] OR "nasal high-flow oxygen therapy” [tiab] OR "high flow nasal oxygen therapy” [tiab] OR "high-flow oxygen therapy” [tiab] OR "nasal high-flow therapy" [tiab] OR "high flow nasal cannula" [tiab] OR "high flow nasal” [tiab] OR "nasal high flow" [tiab] OR "HFNC" [tiab] OR "NHF” [tiab] )AND (Deglutition [MH] OR swallowing [tiab] OR "swallowing reflex" [tiab] OR "swallowing function" [tiab] OR dysphagia[tiab] OR "Deglutition Disorders" [MH] OR "Respiratory Aspiration" [MH] OR "Pneumonia, Aspiration" [MH] OR aspiration [tiab] OR "pulmonary aspiration"[tiab] OR Cough[MH] OR "cough reflex" [tiab])
CINAHL	(("high flow nasal cannula oxygen therapy”) OR ("nasal high-flow oxygen therapy”) OR ("high flow nasal oxygen therapy”) OR ("high-flow oxygen therapy”) OR ("nasal high-flow therapy") OR ("high flow nasal cannula") OR ("high flow nasal”) OR ("nasal high flow") OR ("HFNC") OR ("NHF”) )AND ((MH Deglutition) OR (swallowing) OR ("swallowing reflex") OR ("swallowing function") OR (dysphagia) OR (MH "Deglutition Disorders") OR (MH "Respiratory Aspiration") OR (MH "Pneumonia, Aspiration") OR (aspiration) OR ("pulmonary aspiration") OR (MH Cough) OR ("cough reflex"))
Web of Science	(TS= (high flow nasal cannula oxygen therapy) OR TS= (nasal high-flow oxygen therapy) OR TS= (high flow nasal oxygen therapy) OR TS= (high-flow oxygen therapy) OR TS= (nasal high-flow therapy) OR TS= (high flow nasal cannula) OR TS= (high flow nasal) OR TS= (nasal high flow) OR TS= (HFNC) OR TS= (NHF)) AND (TS= (Deglutition) OR TS= (swallowing) OR TS= (swallowing reflex) OR TS= (swallowing function) OR TS= (dysphagia) OR TS= (Deglutition Disorders) OR TS= (Respiratory Aspiration) OR TS= (Pneumonia, Aspiration) OR TS= (aspiration) OR TS= (pulmonary aspiration) OR TS= (Cough) OR TS= (cough reflex))
Cochrane Central Register of Controlled Trials (CENTRAL)	(("high flow nasal cannula oxygen therapy”):tiab OR ("nasal high-flow oxygen therapy”):tiab OR ("high flow nasal oxygen therapy”) :tiab OR ("high-flow oxygen therapy”):tiab OR ("nasal high-flow therapy"):tiab OR ("high flow nasal cannula"):tiab OR ("high flow nasal”) :tiab OR ("nasal high flow"):tiab OR ("HFNC"):tiab OR ("NHF”) :tiab) AND (MH: [Deglutition] OR (swallowing) :tiab OR ("swallowing reflex"):tiab OR ("swallowing function"):tiab OR (dysphagia) :tiab OR MH: ["Deglutition Disorders"] OR MH: ["Respiratory Aspiration"] OR MH:["Pneumonia, Aspiration"] OR (aspiration) :tiab OR ("pulmonary aspiration"):tiab OR MH: [Cough] OR ("cough reflex"):tiab)

Study Screening and Selection

Titles and abstracts of all studies were independently screened by two of the eight reviewers. The criteria for inclusion were as follows: (1) population: adult patients or healthy participants; (2) concept: dysphagia, aspiration, and pneumonia; (3) context: receiving HFNO; (4) type of paper: any article describing the swallowing function in patients receiving HFNO or the incidence of pneumonia compared with other therapies; (5) language: unrestricted; and (6) publication date: unrestricted. The exclusion criteria were as follows: publication type: reviews, case reports, opinion pieces, qualitative studies, books, letters, oral presentations, posters, and studies for which only an abstract was available. Disagreements between the two reviewers were resolved through discussion, and, if necessary, a third person was brought in for arbitration.

Data Collection and Charting

Data extraction was independently performed by two of the eight reviewers and involved details such as author names, publication year, journal, language, country, research aims, study design, intervention methods, target population, number of participants, age, sex, intubation status, history of dysphagia or aspiration, and outcome measures. Outcome data included HFNO settings and conditions (flow rate of high-flow oxygen therapy, FiO_2_, temperature, and duration), association of high-flow oxygen therapy with aspiration and aspiration pneumonia, and effect of high-flow oxygen therapy on swallowing function. The following characteristics, details, and results were extracted and organized separately for studies involving healthy participants and patients with diseases: (1) authors and publication year, (2) country, (3) study design, (4) sample characteristics, (5) intubation, (6) research aim, (7) intervention, and (8) outcome measures related to swallowing or pulmonary complications. All work was performed collaboratively using online documents, platforms, and cloud services.

Results

**Figure 1:**
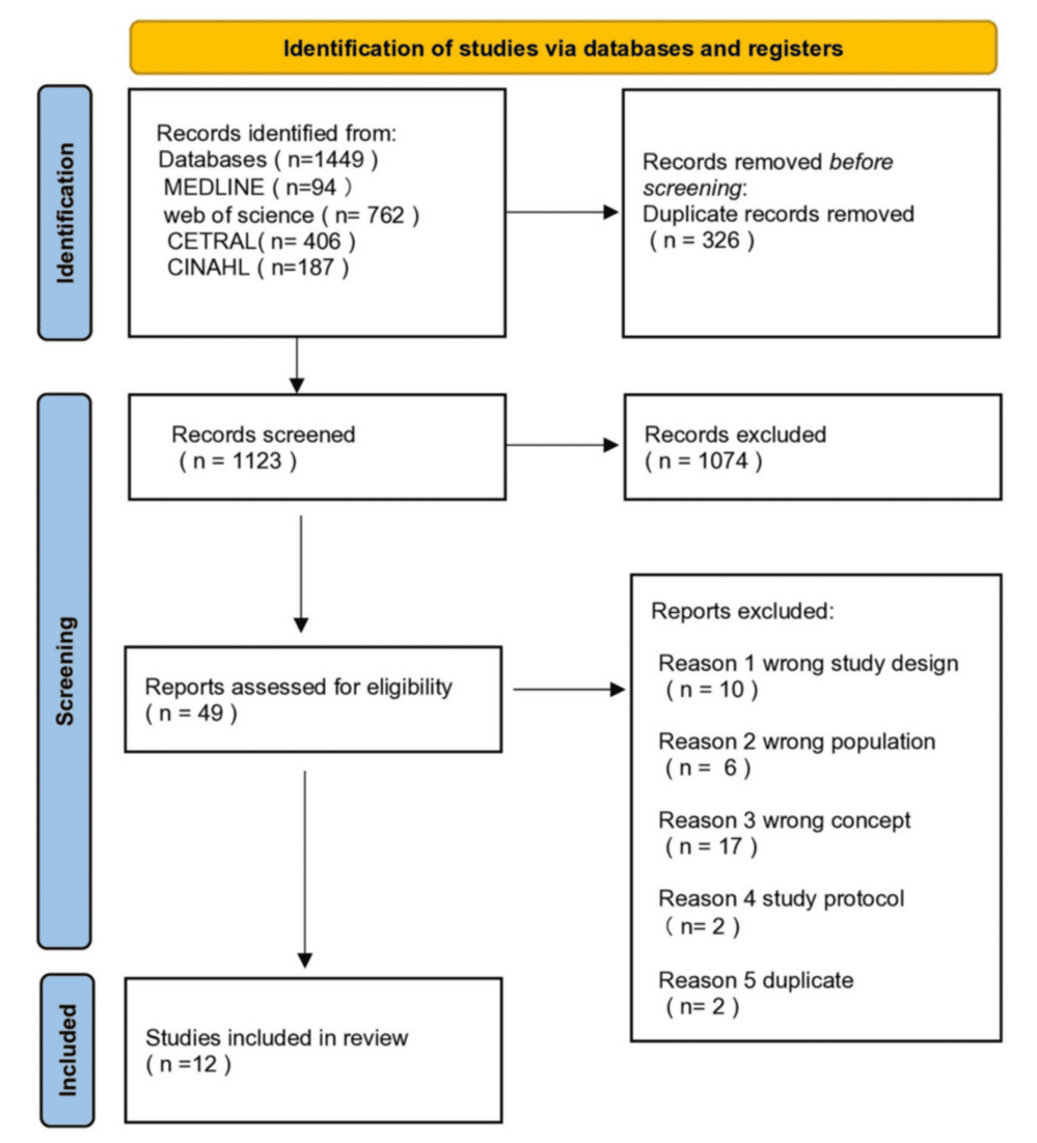
PRISMA 2020 flow diagram depicting the selection of studies PRISMA: Preferred Reporting Items for Systematic Reviews and Meta-Analysis

The literature search, comprising both database and manual methods, yielded 1449 records. We did not apply any language restrictions and non-English papers were also searched for; however, only English-language papers were included in the final version. After removing duplicates, 1123 entries were selected for initial screening. Based on the title and abstract evaluation, 49 articles meeting the inclusion criteria were chosen for further review. After thoroughly examining the full texts, 37 papers were excluded, leaving 12 studies for final analysis. Figure 1 illustrates the study selection procedure.

Characteristics of the Studies

The study designs of the selected studies were as follows: randomized controlled trial (n=3) [14-16], experimental study (n=3) [10,17,18], prospective cohort study (n=2) [4,5], retrospective analysis (n=2) [11,19], prospective randomized interventional 2×2 cross over study (n=1) [12], and multicenter prospective intervention trial (n=1) [20]. The countries in which the studies were conducted were as follows: USA [10,12,17,19,21], Japan [11,18], China [20,22], Egypt [14], and Thailand [15]. The participants were healthy adults in five studies [10,11,17-19] and ill individuals in seven studies [12,14-16,20-22]. The age of the healthy population ranged from 20 to 50 years and that of the population with a medical history ranged from 50 to 90 years.

The review involved a total of 711 participants. Six studies investigated the effects of flow rate on the swallowing function: five involving healthy adults [10,11,17,18,19] and one study involving patients [12]. Almost all the studies used scores to evaluate changes in swallowing function and the presence or absence of aspiration for each flow rate. The outcome measures used were the following validated tools: water swallowing test (WST), repetitive saliva swallowing test (RSST), modified barium swallowing impairment profile (MBSImP), penetration-aspiration scale (PAS) score, and duration of laryngeal vestibule closure (dLVC). Five studies investigated swallowing function [10,11,17-19], in which three used videofluorography (VF) [17,19,21] and one used videoendoscopy (VE) [10]. Four studies reported the incidence of pneumonia associated with other therapies (Table 2) [14,16,20,22].

**Table 2:** Characteristics of the included studies ASA: American Society of Anesthesiologists; dLVC: duration of laryngeal vestibule closure; EMG: electromyogram: FiO_2_: fraction of inspired oxygen; HFNO: high-flow nasal oxygen therapy; ICU: intensive care unit; MBSImP: modified barium swallowing impairment profile; MBSS: modified barium swallow study; PaO_2_: partial pressure of oxygen; PAS: penetration-aspiration scale; RCT: randomized controlled trial; RSST: repetitive saliva swallowing test; SpO_2_: oxygen saturation; VAS: visual analog scale; VE: videoendoscopy; VF: videofluorography; WST: water swallowing test

Study	Study design	Sample characteristics	Post extubation	Research aim	Intervention	Outcome measures related to swallowing or pulmonary complications
Healthy adults						
Sanuki et al. (2017), Japan [18]	Experimental study	9 healthy adults	No	To test the effect of HFNO on the swallowing reflex	Swallowing under different HFNO airflow conditions (0, 15, 30, 45 L/min)	Latency times of the swallowing reflex using EMG. Total number of swallows
Eng et al. (2019), USA [19]	Prospective cohort study	80 healthy adults	No	To test the hypothesis that the use of HFNO negatively affects swallowing performance on objective swallow examination	Swallowing under different HFNO flow rates (20, 40, 60 L/min).	MBSImP using VF
Allen and Galek (2021), USA [17]	Experimental study	29 healthy adults	No	To investigate the influence of airflow delivered via HFNO on the dLVC and describe airway invasion during airflow delivered via HFNO	Swallowing under different HFNO flow rates (0, 10, 20, 30, 40, 50, 60 L/min)	dLVC, PAS using VF
Arizono et al. (2021), Japan [11]	Prospective cohort study	30 healthy adults	No	To assess the impact of HFNO different flow rates on different characteristics of swallowing	Swallowing under different HFNO flow rates (0, 10, 20, 30, 40, 50 L/min)	WST, RSST, and VAS score for swallowing effort during the WST
Graf et al. (2024), USA [10]	Experimental study	27 healthy adults	No	To assess the swallowing function and safety using flexible endoscopic evaluation of swallowing, during HFNO administration at various flow rates	Swallowing tests with different textures under different HFNO flow rates (0, 30, 40, 50, 60 L/min)	PAS using VE
Patients						
Brainarda et al. (2017), USA [16]	RCT	44 patients scheduled for admission to the ICU after thoracic surgery	Yes	To compare the postoperative pulmonary complications in the prophylactic use of HFNO and conventional oxygen therapy	HFNO group: received HFNO at 40L/min. Control group: received the usual nasal cannula or face mask oxygen	Pulmonary complications Discomfort with the HFNO
Yu et al. (2017), China [20]	Multicenter, prospective intervention trial	110 patients underwent thoracoscopic lobectomy for lung tumors	Yes	To compare the reduction of hypoxemia and postoperative pulmonary complications in HFNO and conventional oxygen therapy	HFNO group: received a flow rate of 35–60 L/min and FiO_2_was titrated (45–100%). Control group: received oxygen via either nasal prongs or facemask with oxygen flow titrated (45–100%)	Suspected pneumonia
Flores et al. (2019), USA [21]	Retrospective analysis	10 patients underwent MBSS while wearing HFNO	Yes	This study puts forth clinically relevant observations from a patient population on HFNO and considerations in clinical decision-making about the initiation of safe oral alimentation	Swallowing under different HFNO flow rates (30, 40, 50 lpm)	MBSImP, PAS using VF
Pibul et al. (2021), Thailand [15]	RCT	67 patients with open chest on-pump cardiopulmonary bypass cardiac surgery	Yes	To compare the reintubation rates of patients receiving prophylactic HFNO within 24 h and those receiving incentive spirometer with breathing exercises after cardiac surgical extubation	HFNO group: received 24-h HFNO. Control group: performed deep breathing exercises using an incentive spirometer	Reintubation
Rattanajiajaroen and Kongpolprom (2021), USA [12]	Prospective, randomized, interventional, 2 × 2 crossover study.	22 patients aged 18–80 years, who had been intubated for more than 48 h and had been extubated within the preceding 48 h	Yes	To compare the swallowing-breathing coordination during continuous water infusion between HFNO and low-flow oxygen therapy	Swallowing 10 ml of water in 1 min for 3 times. HFNO group: 50 L/min. Control group: 5 L/min	Number of swallows. Timing of swallowing
Wang et al. (2021), China [22]	Retrospective	283 patients who were hospitalized for serious neurological diseases and were receiving oxygen therapy	Yes	To investigate the role of HFNO in pulmonary complications in critically ill patients with neurological diseases	HFNO group: oxygen concentration and flow rate were adjusted according to the level of PaO_2_ and SpO_2_. Control group: oxygen concentration was adjusted according to the level of PaO_2_ and SpO_2_	Pneumonia
Soliman and Hadidy (2022), Egypt [14]	RCT	80 patients scheduled for major elective upper abdominal surgery, aged 50–70 years, with ASA physical status I–III	Yes	To compare pulmonary complications within 5 postoperative days in HFNO and simple face mask oxygen	HFNO group: the flow rate was adjusted according to the level of SpO2_,_ starting at 35 L/min. Control group: the flow rate was adjusted according to the level of SpO_2,_ starting at 6 L/min	Pneumonia

Effects of HFNO on Swallowing Function

The effects of inspiratory flow rate on swallowing function were categorized as follows: (1) swallowing-breathing coordination, (2) number of swallows, and (3) swallowing function (Table 3).

**Table 3:** The effects of HFNO on swallowing function MBSImP: quantitative assessment of physiological impairment of swallowing function; PAS: determines the severity of laryngeal intrusion and aspiration; RSST: the participants swallow saliva as many times as possible for 30 seconds in a seated position dLVC: duration of laryngeal vestibule closure; E swallow: expiratory swallow; E-I swallow: expiratory-inspiratory swallow; HFNO: high-flow nasal oxygen; I swallow: inspiratory swallow; I-E swallow: inspiratory-expiratory swallow; IQR: Interquartile range; MBSImP: modified barium swallow impairment profile; PAS: penetration-aspiration scale; RSST: repetitive saliva swallowing test; SD: standard deviation; SE: standard error of the mean

Study	Outcome measure	0	≤10	≤20	≤30	≤40	≤50	≤60	Effect on swallowing function
Swallowing-breathing coordination									
Sanuki et al., 2017 [18]	Respiratory rate, times/min, median (10–90th percentile range)	15 (9–17.2)		13 (9.6–14.4)	8 (6.8–15.2)		10 (8.2–13.4)		Respiratory rate decreases as flow rate increases
	Total swallow, n (%)	31 (100)		36 (100)	32 (100)		28 (100)		The high flow rate allowed swallowing in E-I swallow
	I swallow, n (%)	1 (3.2)		4 (11.1)	5 (15.6)		3 (10.7)		
	E swallow, n (%)	27 (87.1)		25 (69.4)	22 (68.8)		18 (64.3)		
	I-E swallow, n (%)	3 (9.7)		7 (19.4)	4 (12.5)		3 (10.7)		
	E-I swallow, n (%)	0 (0)		0 (0)	1 (3.1)		4 (14.3)		
Rattanajiajaroen and Kongpolprom, 2021 [12]	Total swallow numbers, median (IQR)		18.5 (15, 22)				21 (17, 24)		HFNO had a higher percentage of the E-swallow pattern and a lower percentage of the I-swallow pattern
	I swallow number, median (IQR)/%		2.5 (1, 4)/14.4				4.0 (3, 6)/23.1		
	E swallow number, median (IQR)/%		14.0 (9, 21)/74.3				13.5 (11, 19)/67.6		
	I-E swallow number, median (IQR)/%		0.5 (0, 2)/1.1				1.0 (0, 2)/6.1		
	E-I swallow number, median (IQR)/%		1.0 (0, 2)/7.5				1.0 (0, 2)/4.5		
Number of swallows									
Arizono et al., 2021 [11]	Number of swallows, mean (SD)	10.7 (3.5)	10.2 (3.4)	9.1 (3.1)	8.7 (3.0)	8.1 (2.9)	6.8 (2.8)		In RSST, increasing the flow rate decreases the frequency of swallowing
Swallowing function									
Arizono et al., 2021 [11]	Choking, number	0	0	1	0	5	5		Coughing or choking was observed at 40 L/min and 50 L/min
Eng et al., 2019 [19]	MBSImP scores: mean (SE)	8.93 (0.31)		8.90 (0.33)		9.26 (0.34)		10.11 (0.39)	Participants had higher total MBSImP scores at a flow rate of 60 L/min. Significant effect of flow rate for “tongue control during oral bolus hold” and “oral residue“
Sanuki et al., 2017 [18]	Latency times of the swallowing response, mean (SD)	11.9 (3.7)		9.8 (0.9)	9.0 (2.7)		8.5 (3.0)		The latency of the swallow reflex is shorter
Allen and Galek, 2021 [17]	dLVC: mean (SD), [95% CI]	0.36 (0.25), [0.31–.040]	0.36 (0.23), [0.32–0.41]	0.36 (0.21), [0.32–0.39]	0.38 (0.27), [0.33–0.43]	0.39 (0.22), [0.35–0.43]	0.45 (0.33), [0.39–0.51]	0.49 (0.39), [0.42–0.56]	When airflow increases, dLVC also increases
	Frequency of PAS1, count	24	24	19	25	22	19	21	Change in airflow via HFNO is not associated with a change in airway invasion
	Frequency of PAS2, count	121	118	124	120	121	125	122	
	Frequency of PAS3, count	0	2	1	0	2	1	2	
	Frequency of PAS4, count	0	1	1	0	0	0	0	
Graf et al., 2024 [10]	Unsafe PAS (≥6), count (%)	1 (4)			1 (4)	1 (4)	0 (0)	4 (15)	Three sips of thin liquid swallows at 60 LPM are unsafe, and a mean PAS score is higher
	PAS score: mean (SD)	2.3 (1.6)			2.8 (1.7)	2.7 (1.7)	2.6 (1.4)	3.3 (2.0)	

In the swallowing-breathing coordination of a healthy population, the expiratory swallow (E-swallow) is the most common and safe swallowing pattern. However, a higher flow rate significantly decreases the E-swallow pattern and increases the inspiratory swallow (I-swallow) pattern in post-extubation patients [12]. E-I swallowing increases in healthy participants [18]. This suggests that higher flow rates affect the swallowing patterns. Assessment using the 30-mL WST and RSST under each flow condition demonstrated that the frequency of swallowing decreased as the inspiratory flow rate increased, and choking or coughing was observed at 40 L/min and 50 L/min [11].

The latency time of swallowing response decreased with increasing inspiratory flow rate. Particularly, the response time was the shortest at the maximum flow rate (60 L/min) [18]. The MBSImP scores increased as the flow rate increased [19]. The highest scores were recorded at 60 L/min, indicating its possible influence on tongue control and oral residue assessment. The duration of dLVC also tended to increase as the inspiratory flow rate increased [17]. The results indicated that inspiratory flow rate may affect the duration of laryngeal vestibule closure. Regarding the PAS score, changes in inspiratory flow rate did not affect airway penetration. However, under the 60 L/min condition, PAS scores increased in some participants, potentially increasing the risk of liquid aspiration [10]. The frequency of choking or coughing was observed at 40 L/min and 50 L/min [11], suggesting that a high flow rate inhibits the induction of swallowing.

Comparison of Pneumonia Incidence Rate Between HFNO and Conventional Oxygen Therapy

Four studies compared pneumonia incidence rates between HFNO and conventional oxygen therapy (Table 4) [14,16,20,22]: three RCTs [14,16,20] and one observational study [22]. The participants were patients admitted to the ICU after thoracic surgery [16], those undergoing planned thoracoscopic lobectomy [20], those admitted to the ICU with severe neurological diseases [7], and those undergoing elective upper abdominal surgery [15]. The oxygen concentration and flow rate were adjusted to maintain the SpO_2_ above a certain standard. All studies showed that the incidence of pneumonia was not significantly different between HFNO and conventional oxygen therapy. Furthermore, none of the studies analyzed the risk factors for aspiration pneumonia in patients receiving HFNO.

**Table 4:** Comparison of pneumonia incidence between HFNO and COT COT: conventional oxygen therapy; FiO_2_: fraction of inspired oxygen; HFNO: high-flow nasal oxygen; ICU: intensive care unit; PaO_2_: partial pressure of oxygen; RCT: randomized controlled trial; SpO_2_: oxygen saturation

Authors, year	Country	Study design	Population	HFNO	COT	Pneumonia
Brainard et al., 2017 [16]	US	RCT	Patients undergoing thoracic surgery with scheduled admission to the ICU postoperatively	O_2_ at 40 L/min, with FiO_2_ titrated to maintain SpO_2_ ≥90%	The usual nasal cannula or face mask oxygen, titrated by nurses to maintain SpO_2_ ≧90%	HFNO: 1/18 (5.6%), COT: 2/26 (7.7%); p=0.638
Yu et al., 2017 [20]	China	RCT	Patients undergoing planned thoracoscopic lobectomy for lung tumors	A flow rate of 35 to 60 L/min and FiO_2_ is titrated (from 45% to 100%) by the treating clinician to maintain a SpO_2_ of 95% or more	Nasal prongs or facemask with oxygen flow titrated (from 45% to 100%) by the bedside clinician to maintain a SpO_2_ of 95% or more	HFNO: 2/56 (3.6%), COT: 2/54 (3.7%); p=1.000
Wang et al., 2021 [23]	China	Observational study	Patients admitted to the ICU with serious neurological disease and who received oxygen therapy	The oxygen concentration and gas-flow rate are adjusted according to the level of PaO_2_ and SpO_2_, which are maintained at 85–100 mm Hg (PaO_2_) and 95–100% (SpO_2_). The gas temperature is set as 37 °C	The oxygen concentration is adjusted to keep PaO_2_ at 85–100 mm Hg and SpO_2_ at 95–100% with a nasal catheter and mask	HFNO: 9/164 (5.5%), COT: 14/119 (11.8%); p=0.056
Soliman and Hadidy, 2022 [14]	Egypt	RCT	Patients scheduled for major elective upper abdomen procedures	Starting with a flow rate of 35 L/min and temperature of 31 °C, the flow is titrated up to 60 L/min with a target SpO_2_ of ≥94%	A simple oxygen face mask is applied to the patients, starting with a flow rate of 6 L/min, and, titration of a flow rate up to 10 L/min is done to target peripheral oxygen saturation of ≥94%	HFNO: 1/40 (2.5%), COT: 5/40 (12.5%); P=0.201

Discussion

Summary of Evidence

This scoping review identified 12 articles investigating the effects of HFNO on swallowing. Six studies investigated the impact of HFNO on swallowing function, while six examined the occurrence of aspiration pneumonia in patients using high-flow oxygen devices. Four studies on swallowing function used VF or VE, with PAS [9,16] and MBSImP [19] serving as indicators. A higher flow rate of HFNO decreased the respiratory rate, number of swallows, and latency times of the swallowing response, and increased the duration of laryngeal vestibule closure. In contrast, in swallowing-breathing coordination, I swallowing increased slightly, caused choking, PAS scores increased, and the likelihood of aspiration increased. Particularly, thick liquid or puree did not increase the risk of aspiration, but for liquids, choking or coughing increased at inspiratory flow rates above 40 L/min. In the four studies that compared the pneumonia incidence rate between HFNO and conventional oxygen therapy, HFNO did not increase the incidence of pneumonia compared to conventional oxygen therapy. However, it was unclear which patients were at risk for aspiration with HFNO.

Increased flow has both positive and negative effects on the swallowing function; however, its effect on the swallowing function in patients is unclear. Similar results were obtained in a previous review, with positive and negative effects on swallowing function [23]. The extension of laryngeal vestibular closure time and shortening of swallowing latency time have been reported [17,18]. In patients who have been intubated for more than 24 hours, the latency of swallowing may be extended one to two days after extubation [24], which may be effective in patients after extubation. However, this study was conducted on healthy participants, and its efficacy in patients with dysphagia remains unknown.

Exhalation-swallowing-exhalation was the most common breathing pattern during swallowing, followed by inspiration-swallowing-exhalation [25]. As the flow rate of HFNO increased, the inhalation-swallowing-inspiratory pattern increased slightly. These effects may be attributed to the fact that increased airflow increases resistance during exhalation [26], leading to longer exhalation times and increased breath-holding. It was presumed that some participants were more likely to experience aspiration. However, there is scarce research on the effects of endoscopic and fluoroscopic HFNO on the swallowing function in patients. In addition, previous studies have only examined changes in the swallowing function due to differences in flow rates, and the time to use the HFNO therapy was short. It is also necessary to investigate how long-term HFNO use affects swallowing function.

The risk factors for HFNO use in these patients are currently unknown. Case studies have reported the continuous release of vocal cords in the presence of vocal cord paralysis due to recurrent laryngeal nerve palsy [27], which may increase the risk of aspiration. Furthermore, patients intubated for more than 48 hours may develop dysphagia, with a particularly high incidence in patients older than 65 years [28]. Patients with chronic obstructive pulmonary disease (COPD) prone to inspiratory breathing patterns after swallowing [29] may also be at increased risk of aspiration with HFNO use. However, we could not find any studies on patients with vocal cord paralysis or those at risk for dysphagia, such as those with COPD. Further research is needed on patient characteristics that increase the risk of aspiration when using HFNO.

Liquids can cause aspiration; therefore, it may be safer to ingest them at a reduced flow rate; one advantage of HFNO is that it allows food and water intake. This study found that at 60 L/min, there was a risk of aspiration with thin viscous meals. When food is ingested, it is delivered to the pharynx 1.1 s before swallowing begins (range: -0.3 to 6.4 s), and liquids are delivered more quickly [29]. When considering the flow rate in terms of swallowing, we believe that a flow rate lower than 40 L/min is desirable. Choking has been observed during swallowing at 40 L/min, possibly related to greater airway resistance and stress during swallowing. Furthermore, high flow rates (>40 L/min) increase shortness of breath, swallowing time, and dysphagia [30]. Although this study was conducted in healthy participants, a less viscous diet at a flow rate lower than 40 L/min may reduce the risk of aspiration without causing discomfort. The number of references in this study was small, and further studies are required to determine the optimal flow rate for oral intake. In addition, no studies have examined the flow velocity and swallowing function in patients with COPD or acute respiratory distress syndrome, and it is unclear what level of flow velocity is appropriate.

In this review, based on the analysis of actual patient data, no increase in pneumonia was observed. However, it is important to note that this study did not conduct a meta-analysis; therefore, we cannot be entirely certain about this aspect. Additionally, the majority of the participants in the study were postoperative patients, and there is a lack of evidence confirming its efficacy in other disease groups. While the study investigated the incidence of pneumonia, the primary focus was not on aspiration pneumonia or swallowing function. Hence, crucial details such as the amount of water and food consumed by the study participants are missing, and it remains unclear whether the use of HFNO increases the risk of aspiration pneumonia. An observational study found aspiration in four of 39 adult patients with respiratory failure who resumed eating [31]. Going forward, it is essential to investigate patients with COPD [32], patients who may have dysphagia, such as those who have been intubated for more than 24 h, and patients who are orally intubated with high flow.

Strengths and Limitations

This scoping review was conducted based on the current methodological standards in line with PRISMA-ScR guidelines. A comprehensive search, including studies on healthy volunteers and patients with acute and chronic respiratory failure, led to the identification of knowledge gaps and implications for future research on the effects of HFNO on swallowing function. This study has a few limitations. Firstly, most reviews of HFNO on swallowing function have involved healthy participants, and none have examined the effects by direct observation in older patients, extubated patients, or those with impaired swallowing function. Hence, the effect of HFNO on the swallowing function in actual patients is unclear. Second, the duration of invasive ventilation was not taken into account in this study; it is a risk factor for swallowing function and may have affected the results.

Further studies are needed to investigate how the duration of invasive ventilation affects changes in swallowing function with HFNO. In addition, it may be necessary to investigate flow-induced swallowing function in patients at high risk for dysphagia, such as patients with COPD with altered breathing patterns during swallowing and those with long-term intubation. Third, studies investigating the swallowing function using VF or VE are scarce and cannot accurately assess subclinical aspiration. Therefore, some of the findings of this study should be carefully examined. In addition, studies on swallowing function have been conducted in healthy participants. Therefore, the effects of HFNO on patients need to be investigated in more detail in the future. Finally, it is unclear whether changes in swallowing function in patients are caused by HFNO or by the effects or interactions of underlying diseases.

## Conclusions

Our findings showed that increased airflow had both positive and negative effects on swallowing function; however, the actual impact on patients remains unknown. Patient characteristics particularly prone to aspiration when using HFNO may have been involved; therefore, further investigation is warranted. The data from the current study suggested no increase in pneumonia; however, we did not conduct a meta-analysis and hence further studies are required. Additionally, there is also a risk of aspiration during increased airflow and liquid use, but the validation of its efficacy is insufficient, and the risk factors during HFNO use are not clear. Further research investigating patients with possible dysphagia and those with oral intake is warranted.

## Appendices

**Table 5:** PRISMA 2020 Checklist PRISMA: Preferred Reporting Items for Systematic Reviews and Meta-Analysis

Section and topic	Item #	Checklist item	Location where the item is reported
Title			
Title	1	Identify the report as a systematic review	P.1
Abstract			
Abstract	2	See the PRISMA 2020 for abstracts checklist	P.1
Introduction			
Rationale	3	Describe the rationale for the review in the context of existing knowledge	P.1-2
Objectives	4	Provide an explicit statement of the objective(s) or question(s) the review addresses	P.2
Methods			
Eligibility criteria	5	Specify the inclusion and exclusion criteria for the review and how studies were grouped for the syntheses	P.2
Information sources	6	Specify all databases, registers, websites, organizations, reference lists, and other sources searched or consulted to identify studies. Specify the date when each source was last searched or consulted	P.2
Search strategy	7	Present the full search strategies for all databases, registers, and websites, including any filters and limits used	P.2 Table1
Selection process	8	Specify the methods used to decide whether a study met the inclusion criteria of the review, including how many reviewers screened each record and each report retrieved, whether they worked independently, and if applicable, details of automation tools used in the process	P.2
Data collection process	9	Specify the methods used to collect data from reports, including how many reviewers collected data from each report, whether they worked independently, any processes for obtaining or confirming data from study investigators, and if applicable, details of automation tools used in the process	P.2-3
Data items	10a	List and define all outcomes for which data were sought. Specify whether all results that were compatible with each outcome domain in each study were sought (e.g., for all measures, time points, analyses), and if not, the methods used to decide which results to collect	P.2-3
	10b	List and define all other variables for which data were sought (e.g., participant and intervention characteristics, funding sources). Describe any assumptions made about any missing or unclear information	P.2-3
Study risk of bias assessment	11	Specify the methods used to assess the risk of bias in the included studies, including details of the tool(s) used, how many reviewers assessed each study, whether they worked independently, and if applicable, details of automation tools used in the process	P.2-3
Effect measures	12	Specify for each outcome the effect measure(s) (e.g., risk ratio, mean difference) used in the synthesis or presentation of results	P.3
Synthesis methods	13a	Describe the processes used to decide which studies were eligible for each synthesis [(e.g., tabulating the study intervention characteristics and comparing against the planned groups for each synthesis (item #5)]	P.3
	13b	Describe any methods required to prepare the data for presentation or synthesis, such as handling of the missing summary statistics, or data conversions	N/A
	13c	Describe any methods used to tabulate or visually display the results of individual studies and syntheses	P.3
	13d	Describe any methods used to synthesize results and provide a rationale for the choice(s). If meta-analysis was performed, describe the model(s), method(s) to identify the presence and extent of statistical heterogeneity, and software package(s) used	P.3
	13e	Describe any methods used to explore possible causes of heterogeneity among study results (e.g., subgroup analysis, meta-regression)	N/A
	13f	Describe any sensitivity analyses conducted to assess the robustness of the synthesized results	N/A
Reporting bias assessment	14	Describe any methods used to assess the risk of bias due to missing results in a synthesis (arising from reporting biases)	N/A
Certainty assessment	15	Describe any methods used to assess certainty (or confidence) in the body of evidence for an outcome	N/A
Results			
Study selection	16a	Describe the results of the search and selection process, from the number of records identified in the search to the number of studies included in the review, ideally using a flow diagram	P.3 Figure 1
	16b	Cite studies that might appear to meet the inclusion criteria, but which were excluded, and explain why they were excluded	N/A
Study characteristics	17	Cite each included study and present its characteristics	P.3-5 Table 2
Risk of bias in studies	18	Present assessments of risk of bias for each included study	P.3-5 Table 2
Results of individual studies	19	For all outcomes, present, for each study: (a) summary statistics for each group (where appropriate) and (b) an effect estimate and its precision (e.g., confidence/credible interval), ideally using structured tables or plots	N/A
Results of syntheses	20a	For each synthesis, briefly summarise the characteristics and risk of bias among contributing studies	N/A
	20b	Present results of all statistical syntheses conducted. If meta-analysis was done, present for each the summary estimate and its precision (e.g., confidence/credible interval) and measures of statistical heterogeneity. If comparing groups, describe the direction of the effect	N/A
	20c	Present results of all investigations of possible causes of heterogeneity among study results	Table 3 Table 4
	20d	Present results of all sensitivity analyses conducted to assess the robustness of the synthesized results	N/A
Reporting biases	21	Present assessments of risk of bias due to missing results (arising from reporting biases) for each synthesis assessed	N/A
Certainty of evidence	22	Present assessments of certainty (or confidence) in the body of evidence for each outcome assessed	N/A
Discussion			
Discussion	23a	Provide a general interpretation of the results in the context of other evidence	P.8-9
	23b	Discuss any limitations of the evidence included in the review	P.8-9
	23c	Discuss any limitations of the review processes used	P.9
	23d	Discuss the implications of the results for practice, policy, and future research	P.9
Other information			
Registration and protocol	24a	Provide registration information for the review, including the register name and registration number, or state that the review was not registered	P.2
	24b	Indicate where the review protocol can be accessed, or state that a protocol was not prepared	P.2
	24c	Describe and explain any amendments to information provided at registration or in the protocol	N/A
Support	25	Describe sources of financial or non-financial support for the review, and the role of the funders or sponsors in the review	P.12
Competing interests	26	Declare any competing interests of review authors	P.12
Availability of data, code, and other materials	27	Report which of the following are publicly available and where they can be found: template data collection forms; data extracted from included studies; data used for all analyses; analytic code; any other materials used in the review	N/A
